# Tracking implementation within a community-led whole of system approach to address childhood overweight and obesity in south west Sydney, Australia

**DOI:** 10.1186/s12889-021-11288-5

**Published:** 2021-06-26

**Authors:** Nicola Maitland, Karen Wardle, Jill Whelan, Bin Jalaludin, Doug Creighton, Michael Johnstone, Josh Hayward, Steven Allender

**Affiliations:** 1 Health Promotion Service, Population Health, South Western Sydney Local Health District, Liverpool, New South Wales Australia; 2 Global Obesity Centre, Institute for Health Transformation, Deakin University, Geelong, Australia; 3 Population Health Intelligence, South Western Sydney Local Health District, Liverpool, New South Wales Australia; 4 Ingham Institute for Applied Medical Research, University of New South Wales, Sydney, New South Wales Australia; 5 Institute for Intelligent Systems Research and Innovation, Deakin University, Waurn Ponds, Victoria Australia

**Keywords:** Whole of system approach, Implementation science, Childhood obesity, Tracking implementation, Community-based interventions

## Abstract

**Background:**

Obesity is a chronic disease that contributes to additional comorbidities including diabetes, kidney disease and several cancers. Change4Campbelltown implemented a ‘whole of system’ approach to address childhood overweight and obesity. We present methods to track implementation and stakeholder engagement in Change4Campbelltown.

**Methods:**

Change4Campbelltown aimed to build capacity among key leaders and the broader community to apply techniques from systems thinking to develop community-led actions that address childhood obesity. Change4Campbelltown comprised development of a stakeholder-informed Causal Loop Diagram (CLD) and locally-tailored action plan, formation of key stakeholder and community working groups to prioritise and implement actions, and continuous monitoring of intervention actions. Implementation data included an action register, stakeholder engagement database and key engagement activities and were collected quarterly by the project management team over 2 years of reporting.

**Results:**

Engagement activities increased level of community engagement amongst key leaders, the school-sector and community members. Community-led action increased as engagement increased and this action is mapped directly to the primary point of influence on the CLD. As action spread diversified across the CLD, the geographical spread of action within the community increased.

**Conclusions:**

This paper provides a pragmatic example of the methods used to track implementation of complex interventions that are addressing childhood overweight and obesity.

**Supplementary Information:**

The online version contains supplementary material available at 10.1186/s12889-021-11288-5.

## Introduction and background

Obesity is a chronic disease that is an international health priority [1] and contributes to a range of comorbidities including diabetes, kidney disease and several cancers [2]. Obesity prevalence is not evenly distributed with low socioeconomic communities generally experiencing a greater disease burden [3]. Global obesity prevalence has steadily increased over the preceding decades costing an estimated $2 trillion per year, or 2.8% of global GDP [4].

The complex interplay of individual and societal drivers of obesity is a key challenge for prevention, and calls to engage with this complexity as a key part of obesity prevention have existed for a decade [5]. The 2019 Lancet Commission on Obesity pointed to whole of community approaches supported by techniques from systems science as a holding promise to meet this challenge [6, 7], and several current and recent trials of these methods have been undertaken [8]. Bagnall et al. [9] reviewed 33 obesity prevention efforts using a whole of systems approach between 2005 and 2015, reporting instances of favourable behavioural and anthropometric outcomes. The review identified a critical gap in knowledge being how to operationalise, implement and evaluate these whole of systems approaches. The inadequate descriptions of interventions and their implementation represents a significant unmet need in prevention research [10].

Best practice principles exist for the design of community-based prevention [11] including community engagement, program design and planning, evaluation, implementation and sustainability, and governance. Building on these principles are attempts to generalise a ‘process’ that works with community stakeholders to understand the complexity of obesity and deliver evidence-informed and locally relevant prevention activity [12]. Local context is a key element of these interventions, with stakeholder-informed design allowing interventions to differ between communities in direct response to local context - avoiding predefined ‘programs of work’.

While this process of locally-informed prevention design has been operationalised in the context of whole of systems approaches [13], co-creation and the flexibility to alter intervention components between communities provides new challenges for implementation tracking and evaluation. Writing in the Lancet, Rutter et al., critiqued existing approaches to evaluation for relying on pre-determined linear logical models representing inadequate relations of cause and effect and ignoring the complex and dynamic nature of obesity [14]. New tools, such as STICKE (Systems Thinking in Community Knowledge Exchange), are emerging to support communities engaging with complexity [15] by facilitating the visual depiction in a Causal Loop Diagram (CLD) [16]. These diagrams present the underlying logic model for each community and may provide the means to better track program implementation.

In 2017, the Campbelltown - Changing our Future (Change4Campbelltown) [17] initiative brought together key leaders, the school-sector and community members to translate a ‘whole of system’ approach, previously trialled in rural and regional Australian communities, to the Campbelltown Local Government Area (LGA) in south west Sydney, Australia. The initiative began with the development of a stakeholder-informed CLD, reflecting the underlying logic of obesity drivers for the Campbelltown community, and providing the basis for community-led intervention design. In this paper we present a case study for emerging methods to track implementation of actions, and the strength of stakeholder engagement throughout a whole of system approach to address childhood overweight and obesity.

This paper reports on the following research questions:

1. How can the implementation of community-based interventions be tracked over time?
2. How can stakeholder engagement be tracked over time?

## Methods

### Study context

This paper presents analysis and discussion of implementation data collected during the Change4Campbelltown initiative. The intervention aims to build capacity among key leaders and the broader community to apply techniques from systems thinking to develop community-led actions to address childhood obesity. Key components of the intervention include; stakeholder-informed development of CLDs, development of locally-tailored action in response to the drivers of childhood obesity as described in the CLD, formation of key stakeholder and community working groups to prioritise and implement actions, and continuous monitoring of intervention actions. The full protocol for the Change4Campbelltown initiative is published elsewhere [17], and primary and secondary analyses of the main study outcomes will be presented in future publications.

Campbelltown LGA is a large socioeconomically and culturally diverse community situated southwest of the Sydney Central Business District (CBD), spanning across 31,000 ha of land [18]. The current population is 168,000 people, extensive urban development opportunities contribute to the forecasted population growth peaking at 275,800 people by 2036 [19]. Currently, approximately 30% of the population speak a language other than English at home, with the most common languages being Arabic and Bengali [19].

This study has been approved by the South Western Sydney Local Health District Research Ethics Committee (HREC17/LPOOL/314). All methods were carried out in accordance with the study protocol and ethical guidelines and regulations. Informed consent was obtained from all subjects.

### Data sources

#### Participants

Initial participants were drawn from the Campbelltown community, and were actively recruited because of their different levels of authority and influence. Participants were selected to ensure representation across all sectors including local government, non-government organisations, small business, commercial sector, education, community organisations, healthcare providers, cultural groups, resident’s representative groups and sporting organisations.

#### Causal loop diagram

The CLD was developed by local leaders and community stakeholders during three locally facilitated community workshops. During the workshops locally-relevant drivers of childhood obesity were identified, along with the complex, non-linear relationships between those drivers. The resultant diagram served as a logic model underpinning the design of the set of stakeholder-informed activities that comprised the Change4Campbelltown initiative.

#### Implementation records

Implementation data and records were collected, maintained and updated quarterly by the project management team and included an action register, stakeholder engagement database and project management resources including a communication log and Generalised Activity Normalisation Time Table (GANTT) chart.

The action register included information about each distinct action established in the community as part of the initiative. Each action was entered into the register, alongside its primary variable of influence in the CLD. Each action was updated quarterly by the project team, reflecting its status as either emerging but not yet active, active, completed, or abandoned. New actions and additional data on which stakeholders were leading or participating in the action were added to the register. The project team collected action data through face to face conversations, emails, social media, community events and word of mouth. The types of data collection varied, depending on the engagement of the stakeholder leading action, and the type of action they were leading.

The stakeholder engagement database contained records of each stakeholder engaged in the Change4Campbelltown initiative. School-sector stakeholders were defined as any individual who engaged with Change4Campbelltown through engagement or activities in school settings. Community stakeholders were defined as those working or residing in the community, with broad levels of organisational influence represented (ranging from local residents to small business owners). Key leaders were defined as those with positions in the community conferring strong local influence across multiple organisations or sectors (i.e. Mayor, members of parliament, etc.). The number of actions each stakeholder was participating in, or leading was recorded, alongside an assessment of their relative engagement level at each quarter, given by the project manager. The engagement levels used in the action register were based on an adaptation of Rosenblatt’s Engagement Pyramid [20] as it was used in a previous whole systems approach from south-western Victoria [8]. A description of the adapted definitions for the engagement levels is given in Table 1.

**Table 1:** Levels of Rosenblatt’s Engagement Pyramid [20] and adaptation for use in Change4Campbelltown

Engagement Level	Adapted definition
Observing	Sporadic communication with Change4Campbelltown project team. Follows/likes social media accounts, has indicated interest in a network meeting or outreach effort yet has not attended any specific events or provided personal contact details for the purpose of the initiative.
Following	Sporadic communication with Change4Campbelltown project team. Stakeholder occasionally interacts directly with general communications that interest them – such as newsletters, emails, etc.
Endorsing	Knowingly supports action. Understands and supports the initiative without actively contributing to change. Attends events/workshops but shows limited engagement. Commits to short term actions e.g. coming back to another workshop
Contributing	Working on or part of a team working on an action. Attends events and willingly shares related information through networks. Is committed to the cause and passionate about specific action.
Owning	Stakeholder is leading systems change and willingly seeks further opportunity to do so. Understands the approach and will advocate to others for support. Ongoing commitment to Change4Campbelltown, becomes embedded in work/everyday life. Understands joint responsibility ‘we’ instead of ‘you’.
Leading	Acts and advocates to others, for action to make the healthier choice the easier choice but also the wider systems approach. Displays a level of ownership over the initiative and provides feedback for future direction. Stakeholder has evidence that organisational or personal capacity is being directed to engaging new people into Change4Campbelltown activities, and training/building capacity within the initiative.

Numerous ad hoc project manager key engagement activities were conducted as Change4Campbelltown took shape and adapted. Data on the key people engaged, event type (e.g. meeting, festival), the form of engagement (e.g. face to face, digital), the type of organisation (e.g. sporting, education) and community committees joined was collected quarterly using the communication log and GANTT chart (Table 2). These resources were used as references to assist in the assessments of stakeholder engagement captured in the stakeholder engagement database and updating of the action register.

**Table 2:** Change4Campbelltown – Implementation tracking data sources

Data	Description	Date commenced	Collection frequency	Collected from	How
Action register	Overview of all actions, related factor on systems map and theme, status and updates over time	Jul-Sept 2018	Quarterly	Community members, stakeholders & key leaders	Subjective assessment from project manager
Causal Loop Diagram (CLD)	Actions on Change4Campbelltown systems map	2019	Ad Hoc	Action input from action database	STICKE
Stakeholder engagement database	Tracking and assessment of all stakeholders contact details and level of engagement according to engagement pyramid	Sept 2017	Quarterly	Community members, stakeholders & key leaders	Subjective assessment from project manager against engagement pyramid
Communication log	Count of all communication input and output (use of hashtag, dissemination, newsletter subscribers, workshops, community events, generic email)	Jul-Sept 2018	Quarterly	All modes of communication input & output	Objective count
GANTT chart	Overview of grant timeline/reporting requirements mapped against data collection, community action and workshops	June 2017	Ad hoc with funders guidelines	N/A	Updated by project manager according to grant timeline/ reporting requirements

#### Implementation reporting

There is a wide a range of differing approaches to reporting on implementation. The Template for Intervention Description and Replication (TIDieR) [21] checklist was devised by a team of international experts to promote full and accurate descriptions of trial interventions. The original TIDieR template is limited in its ability to report interventions that do not use a randomised control trial study design. The adapted TIDier-PHP template [22] is designed to capture a broader range of interventions including population health and policy interventions. We used this template to describe Change4Campbelltown and this is presented in the supplementary materials (see Additional file 1). This template was not suitable to capture the complexities involved in the Change4Campbelltown intervention due to the rigidity of the pre-designed framework, the inability to capture interventions led dynamically by community members, and concerns for disengagement of community members and stakeholders.

### Analyses

Analysis to track and evaluate the implementation of the Change4Campbelltown initiative was focussed on two complementary processes; evaluating the progression of engagement and active actions over time; tracking the development of recorded actions geographically and against the CLD.

#### Engagement and action strength over time

Using the stakeholder engagement database, the quarterly engagement score of each stakeholder was coded as a numerical score ranging from 1 (observing) to 6 (leading) (Table 1). For each quarter, the total engagement score of all stakeholders was summed. A total engagement score was also calculated for the three stakeholder subgroups separately, indicating total engagement for school, community and key leader stakeholders at each quarter.

#### Action tracking against causal loop diagram

Actions documented in the implementation register were represented on the CLD using built-for-purpose systems mapping software (STICKE). Each action from the register was represented on the CLD and connected with an arrow to its primary point of influence in the diagram. Actions were shown as white boxes with coloured text and outlines to differentiate them from solidly coloured CLD variables. Properties for each action were entered into the software, including its status (active, not active) at each quarter, and which of the themes from the CLD it was primarily connected to (shown by colour-coding the action to match its corresponding theme from the CLD).

#### Geographical distribution of action over time

The geographical location of each active action was recorded in the action database, unless the action operated across the whole Campbelltown area (e.g. council instigating new healthy food policy for council, leisure centres and community events). Each active action was mapped against its geographical location using Google Maps and colour coded by action area (physical activity, healthy eating, education and knowledge and social factors).

## Results

### Engagement and action strength over time

The largest increase in engagement score across the stakeholder group occurred between Q2 and Q3, with an increase of 273 points – concurring with the engagement of 98 additional stakeholders over the same period, and the establishment of the first eight known actions of the initiative (Table 3). There was also large growth in engagement score, stakeholders and action between Q4 and Q5, with 150 additional stakeholders engaged and 19 additional active actions, alongside the second-highest increase in engagement score throughout the initiative (258 points).

**Table 3:** Implementation tracking activities, measures and timeline overview

Activity	Q1	Q2	Q3	Q4	Q5	Q6	Q7	Q8
***Month & Year***	***M18***	***J18***	***S18***	***D18***	***M19***	***J19***	***S19***	***D19***
Workshops	1	2	1	0	1	0	1	0
• Stakeholders at workshops	37	48	102	0	45	0	48	0
Sports organisation engagement	0	0	0	4	6	2	0	1
Key engagement events	0	0	1	4	4	1	3	5
Key community committees joined	0	0	0	3	1	0	0	1
Number of schools engaged for data collection	0	0	0	0	12	0	0	0
• Number of school’s data returned to	0	0	0	0	0	0	0	12
Total number of stakeholders engaged	49	85	183	206	356	357	395	399
• Number of school stakeholders engaged	0	2	12	15	83	83	83	85
• Number of key leaders engaged	24	45	54	59	68	68	77	77
• Number of community members engaged	25	38	117	132	205	206	235	237
Total engagement score of stakeholders	82	188	461	512	770	790	900	916
• Engagement score of school stakeholders	0	4	38	45	151	150	135	135
• Engagement score of key leaders	37	109	143	157	166	172	204	209
• Engagement score of community stakeholders	45	75	280	310	453	468	561	572
Action ideas	0	71	211	0	122	0	0	0
• Active actions	0	0	8	23	42	47	61	63

**Fig. 1:**
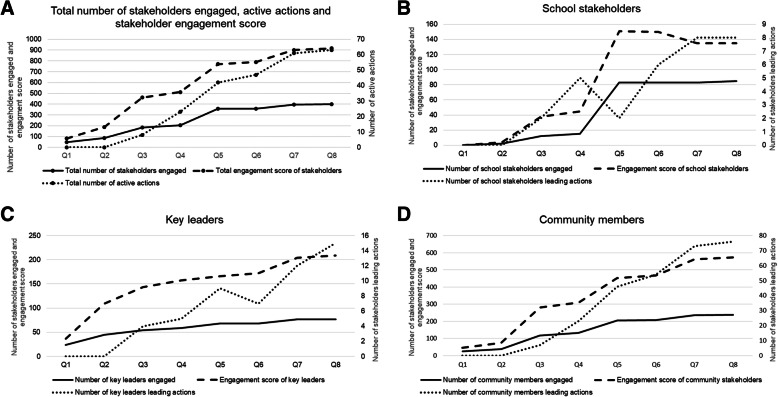
**A** Total number of stakeholders engaged and engagement score plotted against the left vertical axis, and total number of active actions plotted against the right vertical axis. **B-D** Number of stakeholders engaged and engagement score plotted against the left vertical axis, and number of stakeholders leading action plotted against the right vertical axis, by stakeholder category

Figure 1A shows the total number of stakeholders engaged, total engagement score and total active actions at each quarterly time point. The total number of active actions continued to increase with an increase in total engagement score. Total stakeholder engagement score continued to increase as the total number of stakeholders engaged began to plateau. The total stakeholder engagement score reached 916 in Q8 with 399 stakeholders engaged and 63 active actions.

Figure 1B-D shows the growth of school, key leader, community members/stakeholders, their engagement score and the number of stakeholders leading action at each quarter, by stakeholder category. Number of stakeholders leading actions are greater than total active actions (63) due to multiple types of stakeholders collaborating to lead actions at each time point. Community members have the highest engagement score and lead the most active actions. Key leaders were engaged in the initial phase of the two-year period, school stakeholder engagement predominantly occurred in Q4. Actions led by school stakeholders reduced between Q4 and Q5, and plateaued at Q7.

#### Action tracking against causal loop diagram

**Fig. 2:**
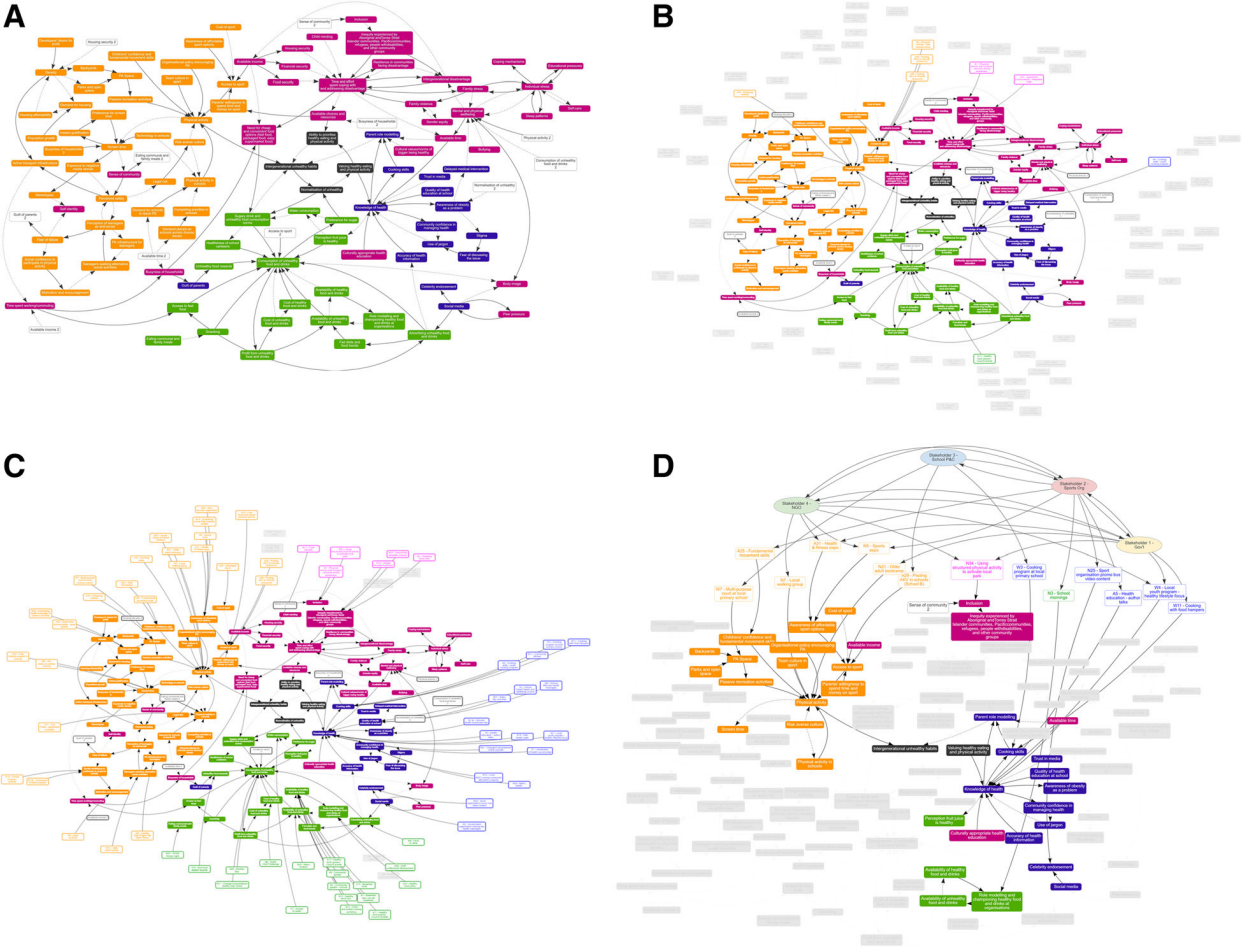
**A** Initial community CLD arranged in four domains: physical activity (orange), education and knowledge (blue), healthy eating (green), social factors (pink). White boxes represent repeated variables located near their relevant domains. **B** and **C** The increasing distribution of actions over time from Q3 to Q8, with actions represented as white boxes with coloured text and border, and non-active actions greyed out. **D** Example of four stakeholder, action and variable relationships on the CLD with stakeholders represented by coloured ovals

The community’s initial CLD identified 106 variables and categorised these into four domains (Fig. 2A). These domains were located around a central core comprised of; valuing and the ability to prioritise healthy eating and physical activity, intergenerational unhealthy habits and normalisation of unhealthy. These domains were; physical activity (orange), education and knowledge (blue), healthy eating (green), social factors (pink).

Figure 2B-C are visual examples of action distribution across primary variables in a CLD at Q3 and Q8, tracked using STICKE software. Figure 2B shows the distribution of active actions at Q3, with the non-active, yet to be developed actions greyed out. Figure 2C shows the distribution and increase of the active actions over time. Active actions increased from eight in Q3 (Fig. 2B) to 63 in Q8 (Fig. 2C). In Q8 there were 25 actions targeting physical activity, 17 targeting healthy eating, 16 targeting education and knowledge and five targeting social factors.

Figure 2D presents a visualisation where four purposely selected stakeholders were added to the diagram, represented by coloured ovals, alongside a sample of their corresponding actions and primary points of impact extracted from the main CLD. The four stakeholders were able to lead 14 different actions across the four domains of the CLD. The sum of variables influenced by action is greater than 14, each action influences more than one variable in the CLD through direct and indirect relationships to other variables. Stakeholders are connected to each other through more than unidirectional relationships.

#### Geographical distribution of action over time

**Fig. 3:**
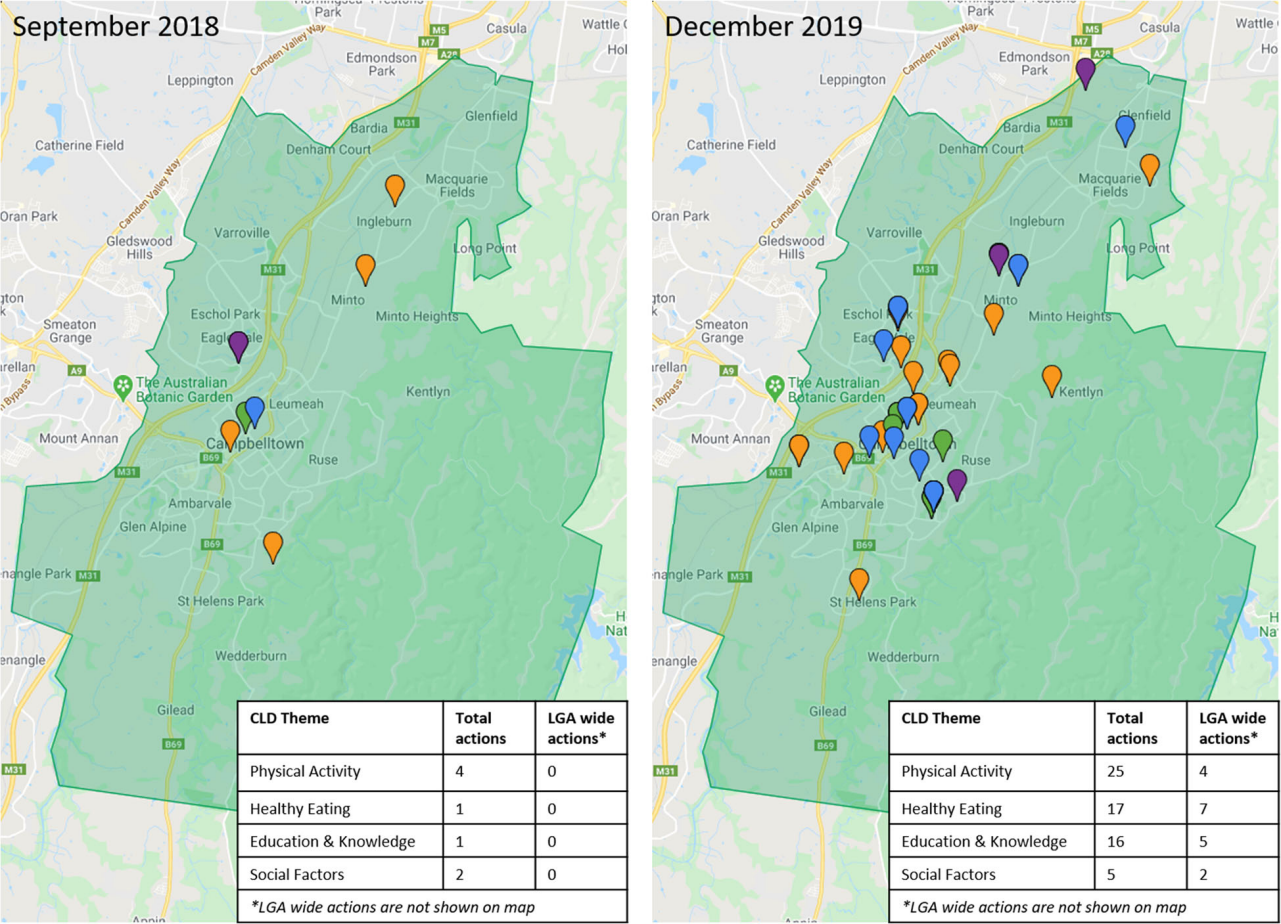
Geographical tracking and distribution of active actions across Campbelltown LGA at two time points (Q3 and Q8). Geographical points are coloured according to theme; physical activity (orange), education and knowledge (blue), healthy eating (green), social factors (pink)

Figure 3 presents geographical distribution of active actions over the 2-year implementation. In Q3 action is clustered around the main CBD with outliers located in school settings. The distribution of actions spread geographically across the LGA over time as action in the physical activity and education and knowledge domains intensified by Q8. Actions predominately occur on the east side of the motorway, increasing in density over time.

## Discussion

### Main findings

This study represents proof-of-concept for emerging methods to track implementation of actions, and strength of stakeholder engagement throughout a whole of system approach to community-based childhood obesity prevention. Initial community and stakeholder engagement was essential in developing the CLD that acted as a base logic model for community-led action and implementation. Key engagement activities conducted by the project team (e.g. community workshops, attendance at community events/festivals, committee membership) enabled the development of strong, sustainable social networks that produced high levels of key leader, school-sector and community member engagement. We observed as engagement increased, the level of community action increased and the community began forming their own networks and developing their own actions with less central support from the implementation team.

Change4Campbelltown was able to demonstrate high levels of stakeholder engagement and with this high numbers of community-led actions were implemented. The levels of engagement appeared to continuously grow but also demonstrate some aspects of seasonality, particularly around school summer vacation (Q4-Q5). With the large number of actions implemented (*n* = 63), there was a range of types of action, types of stakeholder and level of intervention. Actions operated on differing timescales, for many there was some delay between the initial planning and the implementation and following there was often adaptation of the action. Actions were spread across the four domains of the CLD, the physical activity domain had the greatest number of actions addressing primary variables in the CLD at Q8. STICKE was a useful tool to track action against the CLD, with scope for further development of the software to allow for practical community interaction.

The distribution of community-led action across the CLD and geographically across the LGA increased as stakeholder engagement increased. Geographical analyses showed early actions were centrally located relative to the Campbelltown population, spreading throughout the region over time. This was also in parallel to increases in key leader, school and community engagement in terms of number engaged and level of engagement. This mix of formal locations (ie schools and council buildings) and informal settings (ie sports clubs, local community events) also appeared to aid diffusion along the east of the motorway. Used prospectively, these approaches would provide further insight into the distribution of action relative to populations, workplaces and institutions within the community, and strategic targets for future engagement.

### Comparison with other literature and other studies

Following the Lancet Commission for Obesity [6] and the US National Academies of Science, most recently Public Health England have provided guidance to drive whole of system approaches to support promoting healthy weight [23]. In the face of these calls Powell et al., [24] identified the challenges posed to implementation science which included the need to;

(1) enhance methods for designing and tailoring implementation strategies; (2) specify and test mechanisms of change; (3) conduct more effectiveness research on discrete, multi-faceted, and tailored implementation strategies; (4) increase economic evaluations of implementation strategies; and (5) improve the tracking and reporting of implementation strategies. ( [24], pp.1)

This paper is one of the first to tackle several of these challenges in process measurement with sensitivity to complexity. One earlier effort was the WHOSTOPS trial [8] in western Victoria which sought to build capacity of the local community health services, schools and key stakeholders to apply systems science to childhood obesity prevention. The process evaluation [8] parallels this study in identifying the critical nature of the collective effort of stakeholders and particularly institutional relationships in building obesity prevention strategies. The authors point to the adaptive nature of their trial and also to the ways in which the tracking of a broad range of implementation strategies and outcomes was informative to neighbouring prevention efforts.

There are few implementation studies that sought to track large numbers of interventions across multiple stakeholders over time. One exception is Bunger et al., [25] who developed and piloted activity logs completed by key project personnel over an 18-month time period in a multi-context intervention to improve child access to behavioural health services. They collected information about implementation activities, intent, duration and individuals involved reporting on 473 activities within 45 unique strategies. The authors identified that a more nuanced understanding of what it takes to implement different innovations was needed across different phases of implementation, and that this process needed to be able to show how strategies adapt over time.

### Strengths and limitations

The goal of this study was not to scale up an existing program, but to develop an initial set of processes, adaptable to contexts and intervention targets. This represents an extension of the use of systems science in intervention design, which has focussed previously on adaptability, but has yet to develop equivalent structures for implementation tracking over the course of an intervention. The initial process underpinning Change4Campbelltown requires relatively resource-intensive community engagement and network-building activities led by the project team to ensure strong community networks are established to facilitate sustainable community-led action. This resource-intensive community engagement involved significant amounts of face to face time with community members, membership on committees, involvement in community events and ongoing partnership development at all community levels. These processes once established are well suited to fit alongside the switch to strategic leadership engagement that takes place as ownership and leadership of action transfers to the community.

A key challenge with evaluation of community-led action is the ability of the project team to obtain data on actions being implemented relatively independently by stakeholders in the community. This means that the documented number of actions is heavily reliant on relationship and network building between the project staff and community leaders, and in this study means the number of known actions is likely an underestimate. The process also necessarily requires time and resources from the project team and may come at the expense of directly enabling action. This should become progressively less of a concern, however, as ownership of the initiative transfers to community, and the value of insights supporting strategic engagement increases for the project team.

In this study we report data collected by the project team, however stakeholder engagement data was collected and determined solely by the lead project manager. The strength of only one person collecting stakeholder engagement data brings the strength of consistency in data reporting. This approach to data collection is more aligned to the translation of interventions into routine practice where resources for data collection analysis and synthesis are limited to provision of a small number of key project staff. This is an alternate approach to other implementation studies which have multiple project members collecting implementation data [26]. This brings the limitation of only capturing the activities, stakeholders and interactions that are known by and visible to the project team (via adhoc face to face conversations, emails, social media, community events and word of mouth) and so likely underestimates the true scale of the activity.

A further challenge in this study was to identify existing reporting approaches that could adequately track implementation of a complex intervention. We reviewed several reporting templates to assess suitability for this purpose. These included Proctor (2013) [27], which provided a solid implementation science theory base however, the emphasis on reporting ‘strategies’ used in implementation, rather than the tracking of our varied initiatives did not meet our needs. We then reviewed three versions of the TIDieR templates. The original TIDieR [21] was very focused on randomised controlled trials and did not fit well with real-world implementation. The updates to TIDieR proposed by Cotterill (2018) [28] to include elements related to complex interventions was seen as useful and relevant, but had not been taken through the validation required by the EQUATOR network. Due to this, we chose to use the TIDieR-PHP template [22] as it had undergone a rigorous Delphi process and is included within the EQUATOR network of recommended reporting templates. Our TIDieR-PHP [22] case study template is presented in the supplemental materials (Additional file 1). This pre-designed template did not allow for the dynamic nature of community-led action to be accurately captured. Asking community members and stakeholders to fill in templates like these could potentially increase the risk of disengagement, therefore compromising organic community-led action. Using approaches like citizen science in the future will allow community stakeholders to record the implementation of their action from their own perspective, providing greater detail of action implementation from the community perspective.

### Implications for practice

Key to the Change4Campbelltown initiative was repeated, deliberate engagement activities throughout the two-years of implementation. The analyses presented in this study simultaneously demonstrate the success of the approach in driving steady growth in engagement across multiple stakeholder groups, as well as drawing a clear line from that engagement to the establishment of community-led action. There is a critical role for leaders and ensuring they remain engaged in initiatives and the participatory approach to design and tracking of intervention is a key aspect of this engagement.

Prospective use of these methods would offer critical insights about the emergence and focus of action, and the growth in stakeholder engagement in real time. In turn, these methods could support continuous evaluation of implementation, and insights into opportunities to extend community action to new or complementary targets by strategically engaging new stakeholders or attempting to strengthen engagement among existing stakeholders in relevant parts of the community.

The adaptability of the Change4Campbelltown initiative over time was an important aspect of the success of the implementation. As a result the team understood and responded to the community whilst implementing processes to better suit the community in close to real time. The interplay of formal process and organic activities made the initiative more accessible to a wide range of community, increasing engagement and ownership. In translating to other communities the adaptability of process is a legitimate intervention approach that enables this approach to scale to the 168,000 strong Campbelltown community.

### Future research questions

Research is quickly embracing the need for intervention strategies to be tailored to the intended context to enhance outcomes [26]. Gaps remain in our knowledge about how different strategies interact with context and address barriers to implementation. A lack of measures is a commonly cited reason for this gap and the current study has collected additional data, including community readiness for change, environmental audit and children level data, which may provide more insight and which form the broader research associated with Change4Campbelltown.

The Change4Campbelltown CLD has been a critical tool for the project team to measure the distribution and implementation of community-led interventions. The project team also utilised the CLD as a visual tool to initiate conversations with community members and stakeholders when they first became engaged with Change4Campbelltown. A more formal evaluation and analysis of the timing and utility of the CLD will provide valuable information to guide future complex interventions.

Stakeholder engagement is a key element of community-based interventions. The engagement score in this study was collected by one person (project manager). Future research and data collection processes might include multiple people assessing engagement score against the engagement pyramid to allow for the assessment of inter-rater reliability.

Future research should build on this approach to implementation tracking and reporting to develop a complementary statistical analysis of the implementation. More emphasis needs to be placed on tracking the way relationships between leaders engage new activities and strengthen interventions. Methods from social network analysis [29] and agent-based modelling [30] have been trialled in previous studies and should be considered to support future interventions.

## Conclusion

High levels of engagement are critical to facilitate community-led intervention when using a whole of system approach. This paper provides a pragmatic example of the methods used to track implementation of complex interventions that are addressing childhood overweight and obesity.

## Supplementary Information

**Additional file 1.**

## Data Availability

The datasets used and/or analysed during the current study are available from the corresponding author on reasonable request.

## Abbreviations

CBD: Central Business District
CLD: Causal Loop Diagram
GANTT: Generalised Activity Normalisation Time Table
LGA: Local Government Area
STICKE: Systems Thinking in Community Knowledge Exchange
TIDieR: Template for Intervention Description and Replication
