# Supplementary material for: Tracking implementation within a community-led whole of system approach to address childhood overweight and obesity in south west Sydney, Australia
Source: BMC Public Health. 2021 Jun 26;21:1233. doi: 10.1186/s12889-021-11288-5 (PMC8236147; doi:10.1186/s12889-021-11288-5)
Supplement: Supplementary file 1 — Additional file 1. [file 12889_2021_11288_MOESM1_ESM.docx]

**Additional files**

File name: Additional file 1

Format: PDF

# Title: Supplementary material Supplementary material

**Template for Intervention Description and Replication Checklist for Population Health and Policy interventions (TIDieR-PHP)**^1^

| **Framework** | **Framework response** | **What doesn’t fit** |
| --- | --- | --- |
| 1. **Brief name**   **Description and explanation** | Whole of system approach to address childhood overweight and obesity in a socioeconomically and ethnically diverse urban setting |  |
| 1. **Why?** | Describe the logic, mechanisms or rationale of the intervention clearly linking intervention elements to the expected effect on immediate or longer terms outcomes (or both)  Whole of system approach used to address complex issues, used development of CLD/GMB workshops as the planning process  Immediate outcomes:  Increased community cohesion, development of social networks, improved understanding of childhood overweight and obesity as an issue  Longer term outcomes:  Decreased childhood overweight and obesity, increase in healthy lifestyle changes and positive health behaviours | - Dynamic nature of implementation of intervention post GMB process - Framework only fits structured intervention with a formalized process - Only allows for a simple linear logic model to be described - Does not allow for community-led intervention to be considered - Doesn’t respect complexity of logic model and intervention/implementation |
| 1. **What** | Describe any materials used in the intervention (including any online appendices or URL for further details).  CLD/STICKE  For example:   - informational materials (may include those provided to recipients of the intervention or in training of intervention providers)   Systems training for staff (20staff trained)  GMB and community workshops   - Nature and value of any benefit provided (e.g. cash, voucher, meal)   Meal at workshops ($27/head)   - Any physical resources or infrastructure provided as part of the intervention.   N/A | - Doesn’t account for anything outside the structured GMB process - Doesn’t account for human resources required to build community engagement/support community to deliver community-led intervention |
| 1. **What and How?** | Describe how the intervention was planned, established, and intended to be delivered.  Planned with the TRGS grant and GMB process  Depending on the type of intervention, it may be useful to consider:   - how sources of funding for the intervention and the service providers were obtained, how users were enrolled and the service delivered   TRGS grant $745,900 by NSW Ministry of Health   - how any payments were made or benefits delivered, how qualifying conditions were implemented   Grant payment   - the entity being regulated, the scope of the regulation, permitted level of use; procedures for monitoring or enforcing compliance, and any sanctions for non-compliance   N/A – no compliance required for community-led model   - how people were exposed to the intervention, whether it was provided to individuals or larger populations   Community engagement via word of mouth, written communication (print and digital)   - any underpinning legislation including name, date passed and legislative body   N/A | - Intervention is established and delivered by the community – doesn’t fit into framework - No room to capture human resources required to build community engagement to support community-led approach - Doesn’t capture local Council or key partners - Doesn’t capture any in-kind support provided |
| 1. **Who provided?** | Describe the provider of the intervention, including legal status and powers, field organisations and staff responsible for planning, implementation, monitoring and enforcement (will be detailed in manuscript, insert page numbers here). Where relevant, describe intervention provider expertise and training (general or specific to the intervention)  Community members/leaders, Health Promotion Service/Deakin University involved in planning process | - Community members/leaders are responsible for implementation of the intervention - ~400 people involved – not possible to fit into this framework - There is no “enforcement” of intervention due to community-led approach, the fact that there is no “enforcement” is a strength of the intervention implementation |
| 1. **Where?** | Describe the type of location (e.g. school, community centre) and the geographical scope of the intervention (e.g. national, regional, city-wide).  Campbelltown LGA setting described in manuscript  Where relevant, describe the historical, cultural, socioeconomic, or political background to the intervention  Occurs in all settings across whole LGA |  |
| 1. **When and how often** | Describe when the intervention was implemented, how long it remained in place, and if applicable, the number, duration and scheduling of occasions  2 years and ongoing | - Framework assumes a strict intervention timeline with an end date - Intervention is dynamic, designed to move with community therefore has no strict timeline or scheduled events - Intervention is designed to be sustainable overtime with no end date (strength of intervention) - Fundamental flaw of framework is it assumes all interventions are strictly planned with an end date |
|  | **Variations** |  |
| **8.1 Planned variation** | Describe and provide the reason for any variation or tailoring that was planned or allowed for in the design of the intervention. Examples include differences between locations, geographical areas, population subgroups or over time  No planned variation | - Doesn’t suit our intervention |
| **8.2 Unplanned variation** | Describe and provide the reason for any unplanned variation or modifications in the intervention (e.g. between different locations, geographical areas, population subgroups, or over time) that were made after the intervention commenced  N/A | - Doesn’t suit our intervention |
|  | **Fidelity** |  |
| **9.1** | Describe any strategies used or actions taken to maintain fidelity of the intervention (i.e., to ensure that the intervention was delivered as intended) | - Intervention is designed to highlight the specific context and strengths of individual communities, there is no set intervention/program, therefore this is N/A |
| **9.2** | Describe the fidelity of the intervention (i.e., the extent to which the intervention was delivered as intended)  as above | - As above – framework assumes it is a set/structured intervention with a strict linear model – not suited to our intervention |
| **10 Stage of implementation *(Cotterill et al. 2018)*** | - proof of concept - proof of implementation (how does it work in real-world settings) - implementation at scale - longer term sustainability |  |
| **11 Voice**  **(Cotterill et al. 2018)** | Who was involved in preparing the template?  Project staff (KW, NM), Deakin researchers (SA, JW) |  |

**References**

1. Campbell M, Katikireddi SV, Hoffmann T, Armstrong R, Waters E, Craig P. TIDieR-PHP: a reporting guideline for population health and policy interventions. BMJ. 2018 May 16;k1079.
